# Application of green mussel (*Perna viridis*) shells hydroxyapatite on osteocalcin levels and osteoblast cells in rabbit femur bone defect

**DOI:** 10.1097/MS9.0000000000001302

**Published:** 2023-09-14

**Authors:** Rafika Syah Putra, Nyoman Suci Widyastiti, Selamat Budijitno, Muflihatul Muniroh, Robin Novriansyah, Luqman Alwi, Putu Anda Tusta Adiputra

**Affiliations:** Departments of a Surgical; b Clinical Pathology; c Physiology; d Orthopedic and Traumatology, Faculty of Medicine, Diponegoro University; e Surgical Department, Faculty of Medicine, State University of Semarang, Semarang; f Surgical Department, Faculty of Medicine, Udayana University, Denpasar, Indonesia

**Keywords:** Green mussel shell, Hydroxyapatite, Osteocalcin, Osteoblast cells

## Abstract

**Background:**

Bovine hydroxyapatite (HA) used for bone grafts is relatively expensive, necessitating the development of alternative sources. Alternative HA materials derived from green mussel shells with smaller molecular sizes are inexpensive and abundantly available throughout Indonesian waters. The purpose of this study is to investigate the effect of green mussel shells HA on bone healing.

**Methods:**

This post-test-only experimental research used male rabbits with femoral defects divided into three groups randomly: K (no treatment), P1 (bovine HA treatment), and P2 (green mussel shell HA treatment). The osteocalcin level was assessed biochemically while osteoblast cells were histopathologically at the second, fourth, and sixth weeks. Statistic tests were used to assess differences between groups and periods with statistical significance *P*<0.05.

**Results:**

Nine rabbits in each group showed significant differences between groups K, P1, and P2 in term osteocalcin levels at week 2 (2.60, 4.53±0.12, 4.47±0.23; *P=0.046*), week 4 (5.13±0.12, 8.53±0.12, 7.47±0.12; *P=0.025*), and week 6 (8.20, 11.93±0.23, 10.93±0.31, *P=0.023*), while in term osteoblast cells only at week 6 (16.33±3.46, 26.10±3.52, 30.40±3.29; *P=0.006*). The osteocalcin level and osteoblast increased significantly between groups K and P1/P2 from the initial trial until the last week. Osteoblast cells in the groups P1/P2 increased significantly, especially at week 6.

**Conclusion:**

Green mussel shell HA has the biochemical effectiveness of osteocalcin and can increase osteoblast cells comparable to bovine HA, which can enhance bone healing.

## Background

Highlights

- Hydroxyapatite (HA) derived from bovine tends to be more expensive, has limited production, and is quite expensive, hence an alternative source with comparable efficacy is required.
- Green Mussel (*Perna viridis*) shell is a possible source of HA that is inexpensive and abundantly available throughout Indonesian waters.
- Green mussel shell HA has the biochemical effectiveness of osteocalcin and can increase osteoblast cells comparable to bovine HA which can enhance bone healing.

Fracture healing requires a delicate balance between biological processes and mechanical stability. Bone grafts are commonly utilized to aid in the bone healing process. Autografts, or bone transplant material derived from the same individual body component, have long been considered the gold standard for correcting bone abnormalities.^1–3^ However, the requirement for additional operational procedures and the limited amount of material are areas where this technology could be improved. To address this, other natural materials with comparable benefits, such as bone grafts, are required.

The main inorganic component of bone, hydroxyapatite (HA), is a biomaterial that is frequently used in bone grafting operations. Due to its osteoconductive qualities, HA is a potent material for bone substitutes because it is one of the components of the diamond-like concept that underpins the process of bone repair.^4^ The most often used HA is derived from bovine bone (HA bovine). The manufacturing technique, however, remains complicated, and the price is very high.^5^ As a result, an alternate fundamental material, particularly green mussel shells, is required. Studies reporting the use of green mussel (*Perna viridis*) shells as a potential material for synthesizing HA are the references in this study.

Indonesia produces around 140–210 tons of green mussel shell waste (*Perna Viridis*) per hectare annually, with 95.69% of the total weight of green mussel shells composed of HA.^6^ It is estimated that around 133.97–287.07 tons of HA per hectare are produced annually.^7^ Green mussel shells are a possible option for manufacturing HA due to their abundance, ease of availability, smaller particle size, and higher HA content.

Levels of osteocalcin and osteoblast cells are components used as markers of the bone healing process.^8,9^ Sari *et al*.^10^ studied the use of seashells and sea cucumber on the femoral defects of Wistar rats and found significant differences in the number of osteoblasts and osteoclasts between groups. Thahir *et al*.^11^, who used snakehead fish bone xenografts on periodontal bone defects in marmots, showed increased osteocalcin levels in the treatment group on the 14^th^ and 21^st^ days. Thus, this study aims to prove the effectiveness of Green mussel shell HA as a bone graft material as assessed by levels of osteocalcin and the number of osteoblast cells.

## Method

Laboratory experimental research with a post-test-only control group design was carried out from October 2022 to April 2023. The experiments were conducted following the institutional guidelines for animal treatment, and the protocol was approved by the Health Research Ethics Committee on 27 September 2022 (Protocol Numbers: 113/EC/H/FK-UNDIP/IX/2022) and fully compliant with ARRIVE guidelines.^12^

### Hydroxyapatite preparation

Before HA implantation, it was calculated that 300 mg of HA would be required to implant a tubular defect 5 mm in diameter and 5 mm in depth.

### Animal models and surgical procedures

Male New Zealand rabbits (*Oryctolagus cuniculus*), aged 6–12 weeks and weighing 2.5–3 kg, were used as experimental animal models. There criterias were chosen so that research subjects were considered homogeneous and reduced research bias. Body weight was measured for each rabbit before the study. Then the rabbits were selected according to the inclusion criteria. The sample size is calculated using the resource equation method by calculating the “E” value, where the “E” value must be between 10 and 20, with the following formula^13^:


E=totalnumberofexperimentalanimals−numberofgroups

There were three groups, each of which was assessed three times (at weeks 2, 4, and 6) for a total of nine groups, each consisting of three samples. So the total number of experimental animals was 27. The “E” value (27−9=18) is still in the range of 10 and 20. The number of samples for each group was then added with a 10% dropout, so the number of samples for each group was 4, and the total number of samples was 36. Thirty-six rabbits that met the inclusion criteria were then adapted at 28,0±2,0^o^C room temperature and given food and drink ad libitum for 1 week. During the adaptation period, rabbits were given vitamin ADE (0.1 ml/KGB IM), vitamin B complex (0.1 ml/KGB IM), and ivermectin (0.4 mg/KGB IM) to prevent disease. After the adaptation period, all rabbits were separated into one cage and randomized by the researchers using a computer program into three groups: control group (K), bovine HA graft treatment (P1), and green mussel shell HA graft treatment (P2).

In the surgical procedure, injection of enrofloxacin (5 mg/KGB) intravenously (IV) into the lateral auricular vein in the ear as a prophylactic antibiotic is followed by injection of ketamine (25 mg/KGB) and acepromazine (0.3 mg/KGB) intramuscularly (IM) in the longissimus dorsi caudal muscle as an anaesthetic. In the surgical area that has been shaved and disinfected, an incision is made to reach the periosteum of the rabbit’s femur. A 5 mm diameter and 5 mm deep defect were created using a drill in the lateral aspect of the distal femoral metaphysis. In group P1, the defect was filled with bovine HA; in group P2, it was filled with HA from green mussel shell. After the procedure, suturing is done to close the incision wound. All rabbits were observed for signs of inflammation and activity every 12 hours and given ketoprofen IM (2 mg/KGB) painkillers and enrofloxacin IM (5 mg/KGB) antibiotics every 24 h for 3 days. Rabbits that died during treatment or suffered postoperative infections were excluded. On the day of the examination, samples from each group were taken, and the investigators were only aware of the sample’s identity after the data were collected.

### Outcome evaluation

#### Osteocalcin levels

The biochemical assessment was carried out by measuring the level of the biomarker osteocalcin in rabbit blood serum at the second, fourth, and sixth weeks postoperatively. Osteocalcin examination was carried out using the Mindray BC2800Vet series and Blood Chemistry Analyzer (Ubio-iChem-535),^14^ examined by two clinical pathology specialists with normal reference values of 11.3–25.6 ng/ml according to Hannemann *et al*.^15^

#### Osteoblast cell count

The centre of the treatment area of the necropsied rabbit femur was made up of slides for histopathological assessment at the second, fourth, and sixth weeks postoperatively. The bone healing process is measured based on the number of osteoblast cells. Slides were stained with hematoxylin-eosin for histopathological examination using a light microscope with ×400 magnification in five fields of view. Two Anatomical Pathology specialists performed the histopathological assessment. The investigators in this study only became aware of the identity of the sample.

### Data analysis

The Shapiro-Wilk test was used to assess the normality of the data distribution. If the data is normally distributed, a Paired *t*-test is used to compare the differences between groups each week. The Wilcoxon matched pairs test will be applied if the data are not normally distributed. The one-way Anova test was used to compare differences across groups per week, followed by the post hoc LSD test if the data were normally distributed, otherwise, the Kruskal-Wallis and Mann-Whitney tests were used. Data were analyzed with SPSS version 25 software for Windows 10, with the result considered significant if *P*<0.05.

## Results

All 36 rabbits recovered well from anaesthesia and surgical intervention, and all wounds in the rabbits were healed after surgery. During the trial period, three rabbits in each group died or dropped out due to complications of surgical infection problems.

### Osteocalcin serum levels

The mean osteocalcin levels at 2, 4, and 6 weeks were found to be the highest in group P1, followed by group P2, and lastly group K, with an abnormal data distribution. The Kruskal-Wallis unpaired t-test showed significant differences in osteocalcin levels at 2, 4, and 6 weeks (Table 1). The Mann-Whitney test of osteocalcin serum levels showed a significant difference in the second week between groups K and P1, as well as groups K and P2, but there was no significant difference between groups P1 and P2. In the fourth week, there was a significant difference between groups K and P1 and P2, and there was also a significant difference between groups P1 and P2. In the sixth week, there was a significant difference between groups K and P1 and P2, and there was also a significant difference between groups P1 and P2 (Table 2). The Wilcoxon paired test showed that the entire study group did not have a significant difference (Table 3).

**Table 1:** Baseline characteristics data.

Variable		Group	*N*	Mean±SD	*p*
Osteocalcin level^a^	Week 2	K	3	2.60	0.046^*^
		P1	3	4.53±0.12	
		P2	3	4.47±0.23	
	Week 4	K	3	5.13±0.12	0.025^*^
		P1	3	8.53±0.12	
		P2	3	7.47±0.12	
	Week 6	K	3	8.20	0.023^*^
		P1	3	11.93±0.23	
		P2	3	10.93±0.31	
Osteoblast cell^b^	Week 2	K	3	14.83±4.21	0.630
		P1	3	15.47±3.23	
		P2	3	17.43±2.22	
	Week 4	K	3	16.57±2.80	0.730
		P1	3	15.37±4.63	
		P2	3	14.27±2.57	
	Week 6	K	3	16.33±3.46	0.006^*^
		P1	3	26.10±3.52	
		P2	3	30.40±3.29	

a Kruskal-Wallis.

b One-way ANOVA.

* Significant (*P*<0.05).

**Table 2:** Comparison of each group for osteocalcin level and osteoblast cell.

Variable		P1	P2
Osteocalcin level^a^			
Week 2	K	0.034^*^	0.034^*^
	P1	–	0.796
Week 4	K	0.043^*^	0.043^*^
	P1	–	0.043^*^
Week 6	K	0.034^*^	0.037^*^
	P1	–	0.046^*^
Osteoblast cell^b^			
Week 6	K	0.013^*^	0.002^*^
	P1	–	0.175

a Mann-Whitney.

b LSD.

* Significant (*P*<0.05).

**Table 3:** Comparison of osteocalcin level and osteoblast cells between periods of time.

		Week		
Variable	Group	2–4	2–6	4–6
Osteocalcin level^a^	K	0.102	0.083	0.102
	P1	0.083	0.109	0.109
	P2	0.109	0.102	0.109
Osteoblast cell^b^	K	0.226	0.681	0.924
	P1	0.984	0.025^*^	0.01^*^
	P2	0.080	0.032^*^	0.036^*^

a Wilcoxon paired test.

b Paired *t*-test.

* Significant (*P*<0.05).

### Osteoblast cells

**Figure 1:**
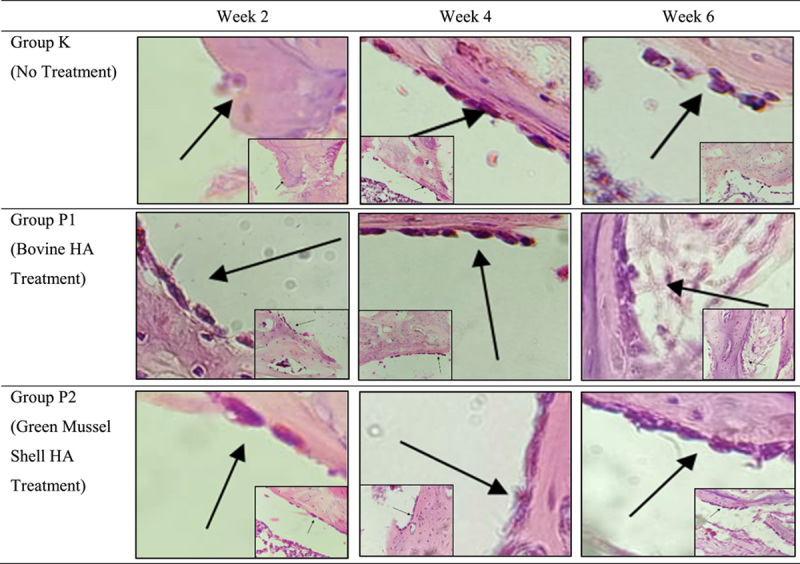
Histopathological picture of rabbit bone in the healing process stained with hematoxylin and eosin. The osteoblasts are blue-painted cell nuclei. The number of osteoblast cells at week 4 increased by a relatively equal number throughout the group. Furthermore, a significant increase in osteoblast cells was observed in week 6 of the treatment groups P1 and P2. HA, hydroxyapatite.

The histopathological picture is presented in Fig. 1. The histopathological examination results at weeks 2 and 6 showed the highest average number of osteoblast cells in group P2. Whereas in week 4, the highest number of osteoblast cells was found in group K. The data were found to be normally distributed, and the one-way Anova test in weeks 2 and 4 showed no significant difference (Table 1). There is a significant difference in the number of osteoblast cells at week 6 (*P*=0.006), so it is continued with the LSD post hoc test.

The post hoc LSD test at week 6 of osteoblast cells showed significant differences between group K and groups P1 and P2, while there was no significant difference between groups P1 and P2 (Table 2). Paired *t*-test of the number of osteoblast cells in group K showed no significant difference. There were significant differences in the P1 and P2 groups between weeks 2 and 6, and between weeks 4 and 6 (Table 3).

## Discussion

This study aims to prove the effect of giving green mussel shells HA on the healing of bone fractures in rabbits with femur defects. The healing of bone fractures in rabbit femur defects was assessed using osteocalcin levels and osteoblast cells. Osteocalcin is a non-collagen protein specifically produced by osteoblast cells to enhance its function in bone healing. The serum osteocalcin level is the main biomarker of bone healing and can be measured using the automated electrochemiluminescence immunoassay method.^11,14^ The progress of bone healing is proportional to the level of osteocalcin. Therefore, higher osteocalcin levels can be assumed to have better bone healing.

This study showed significant differences in serum osteocalcin levels between groups at weeks 2, 4, and 6. Comparisons between each group at weeks 4 and 6 were all significantly different, however, at week 2 significant difference only existed between the control group and the treatment group (P1 and P2). This shows that giving bovine HA and green mussel shell HA can accelerate the process of bone healing when compared to the group without HA administration. This finding is in line with Irianto and colleagues and Thahir and colleagues, which also proved the same.^9,11^ Descriptive data analysis can indicate that in terms of efficacy, bovine HA has slightly better potential for increasing osteocalcin than green mussel shell HA.

Osteoblast cells are mononuclear cells that regulate bone metabolism by synthesizing the bone matrix, which gradually undergoes mineralization and develops into osteocytes.^15,16^ Osteoblast cells can be measured directly through histopathological examination. This study shows that there is no significant difference in the number of osteoblast cells at weeks 2 and 4, but there is a significant difference at week 6. The measurement of group differences at week 6 indicated a significant difference between groups K and P1 and P2, but no significant difference between groups P1 and P2. In the comparative analysis of osteoblast cell counts between periods, significant differences were found at weeks 2–6 and weeks 4–6 in groups P1 and P2. These results indicated that giving bovine HA and green mussel shells HA accelerated bone healing compared to the group without HA. Research by Sari and colleagues also showed that giving HA can significantly increase the number of osteoblast cells, with a study by Lunardhi and colleagues showing the highest number of osteoblast cells on days 3 and 7 after HA administration.^10,17^ Descriptive data analysis showed that the number of osteoblast cells in green mussel shells HA had a slightly higher value than the bovine group, which had higher scores than the control group.

HA has two properties; osteoinduction (increasing osteoblast cell activity through increasing osteocalcin) and osteoconduction (recruiting new osteoblast cells to the bone healing area).^18^ HA will be broken down into the ions needed by bone to carry out healing, namely Ca^2+^ and inorganic phosphate. Both of these compounds will activate osteoblast cells and start the induction process to increase the activity of osteoblasts by increasing osteocalcin. High levels of osteocalcin will act as a conducting agent for other osteoblast cells to come to the lesion site and perform their bone repair duties.^19^ Osteoblast cells synthesize and release bone matrix to maintain structural integrity and bone shape, which promotes formation, remodelling, and bone healing.^20^ Therefore, the use of osteoblast cells as biomarkers can be interpreted as meaning that the higher the value of osteoblast readings in histopathological assessment, the better the bone healing activity in the observed bones.^21^

The experimental model of the fracture healing process in rabbit femurs may simulate the clinical condition of fracture patients as well as interventions conducted on the condition with calibrated doses. This approach can be used to assess the effectiveness of therapy by measuring cellular activity and protein products involved in the fracture healing process.

The study’s limitations are that it did not examine additional variables affecting bone healing, such as osteoclast cells, osteoprotegerin, collagen levels, RANKL, etc. In addition, the sample size in this study was small, so further research should use a larger sample size.

## Conclusion

The osteocalcin biochemical efficacy of green mussel shell HA was close to that of bovine HA, with osteocalcin serum levels in green mussel shell HA slightly lower than bovine HA. Meanwhile, the histopathological efficacy of green mussel shell HA was better than bovine HA, with the histopathological value of green mussel shell HA being higher than bovine HA.

## Ethical approval

The experiment was approved by the Research and Ethics Committee of the Faculty of Medicine Diponegoro University, Indonesia (Protocol Numbers: 113/EC/H/FK-UNDIP/IX/2022).

## Consent

This study used experimental animals. Experiments were conducted following institutional guidelines for animal care with best practice animal treatment during the study, including adaptation period, good feeding and housing, during the procedure as well as post-intervention care that considered the safety of the experimental animals. Details of this statement have been stated in the article manuscript

## Sources of funding

This research did not receive funding from anyone

## Author contribution

R.S.P.: conceptualization, funding acquisition, investigation, methodology, writing—original draft. N.S.W.: conceptualization, data curation, project administration, writing—original draft. S.B.: conceptualization, formal analysis, funding acquisition, validation, writing—review and editing. M.M.: methodology, resources, writing—review and editing. R.N.: supervision, validation, writing—review and editing. L.A.: visualization, formal analysis, investigation, writing—review and editing. P.A.T.A.: supervision, visualization, writing—review and editing.

## Conflicts of interest disclosure

The authors declare no conflicts of interest.

## Research registration unique identifying number (UIN)

This research does not use human subjects.

## Guarantor

Selamat Budijitno.

## Provenance and peer review

Not commissioned, externally peer-reviewed

## Data availability statement

No other datasets were used in this study.

## Acknowledgements

The authors thank Semarang Animal Centre Veterinary Clinic, Diponegoro University Veterinary Laboratory, Semarang, and “Satwa Sehat Indonesia” Animal Clinical Laboratory Division, Malang for the support given during the study.
